# Work–family conflict, emotional exhaustion and performance-based self-esteem: reciprocal relationships

**DOI:** 10.1007/s00420-014-0941-x

**Published:** 2014-03-25

**Authors:** Anne Richter, Karin Schraml, Constanze Leineweber

**Affiliations:** 1 Department of Psychology, Stockholm University, 106 91 Stockholm, Sweden; 2 Stress Research Institute, Stockholm University, 106 91 Stockholm, Sweden

**Keywords:** Emotional exhaustion, Performance-based self-esteem, Self-esteem, Structural equation modelling, Work–family conflict

## Abstract

**Objectives:**

The three constructs of work–family conflict, emotional exhaustion and performance-based self-esteem are all related to tremendous negative consequences for the individual, the organization as well as for society. Even though there are studies that connect two of those constructs, the prospective relations between all three of them have not been studied yet.

**Methods:**

We explored the prospective relations between the three constructs in a large Swedish data set representative of the Swedish workforce. Gender differences in the relations were investigated. Longitudinal data with a 2-year time lag were gathered from 3,387 working men and women who responded to the 2006 and 2008 waves of the Swedish Longitudinal Occupational Survey of Health. Four different cross-lagged models were analysed.

**Results:**

In the best fitting model, higher levels of work–family conflict at time 1 were associated with an increased level of performance-based self-esteem at time 2, but not with emotional exhaustion, after controlling for having children, gender, education and age. Also, relationships between emotional exhaustion at time 1 and work–family conflict and performance-based self-esteem at time 2 could be established. Furthermore, relationships between performance-based self-esteem time 1 and work–family conflict and emotional exhaustion time 2 were found. Multiple-group analysis did not show any differences in the relations of the tested constructs over time for either men or women.

**Conclusions:**

We conclude that the three constructs are interrelated and best understood through a reciprocal model. No differences were found between men and women.

## Background

In modern Western society, chronic stress is a major public health problem as it increases the risk of stress-related ill health and diseases (Perski 2006; Shirom 2003). In Sweden, stress-related health problems, such as emotional exhaustion and clinical burnout, are among the most common diagnoses for long-term sickness leave (Lidwall 2010). One contributing factor to the growing number of these health problems might be the increase in dual earner couples and single parents as these workers may more often face difficulties in organizing work and non-work (e.g. home) responsibilities. Imbalance between work and family demands is often described in terms of ‘stress’ and appears to be a core stressor that erodes well-being (Bellavia and Frone 2005). Also, individuals with a strong need to prove their competence and to exert maximum effort in order to feel worthy, i.e. individuals with high performance-based self-esteem, are at increased risk to suffer from feelings of stress. Although several previous studies have investigated relationships between emotional exhaustion, work–family conflict and performance-based self-esteem, only two of the three components were studied simultaneously. In addition, studies with a longitudinal design are lacking, and at this point in time, we lack a deeper understanding of how these three components are related to one another over time. Moreover, only a few studies have used national representative data, which makes it hard to generalize findings to a broader occupational population. In the present study, we address these research gaps and test the causal relationship of work–family conflict, emotional exhaustion and performance-based self-esteem based on longitudinal data from a large Swedish national representative sample.

To begin with, we will give a definition of the three constructs and then describe their potential relations to each other. The first construct, i.e. *work*–*family conflict* represents a stressor associated with being involved in several roles (i.e. the work role and a role outside work such as mother, father, spouse), where work is predicted to affect the non-work domain negatively. In other words, work–family conflict is prevalent when role pressures from work and family domains are mutually incompatible in some respect (Greenhouse and Beutell 1985). Lack of time and energy due to the double burden of work and home demands might increase feelings of insufficiency and imbalance between the work and the family domain. In Sweden, the number of dual earner couples with both partners working full time is high. Moreover, in a representative Swedish sample, as much as 25 % of all men and 31 % of all women reported work-family conflict at some time during a week (Lidwall 2010) and an international comparison indicated that Swedish men and women experience work–family conflict more often than those in other European countries (Strandh and Nordenmark 2006). It has been frequently reported that work–family conflict is associated with negative consequences that affect both the work and family (Allen et al. 2000; Amstad et al. 2011). Moreover, negative consequences for employees’ health have been well established (Eby et al. 2005).

The second construct, i.e. *emotional exhaustion*, is the most central aspect of burnout and refers to a feeling of being overextended and depleted of one’s emotional and physical resources (Maslach and Leiter 2008). It is suggested to be the first symptom of burnout to develop (Toppinen-Tanner et al. 2002) and can thus be seen as an indicator for chronic stress. Emotional exhaustion occurs when employees experience an emotionally demanding work situation under a longer time period (Schaufeli and Greenglass 2001) and has been related to feelings of frustration and anxiety (Cordes and Dougherty 1993; Pines and Maslach 1980) as well as to negative effects in the work domain (Lee and Ashforth 1993), e.g. deterioration in the quality of service, higher job turnover and absenteeism, and low morale (Brotheridge and Lee 2002; Grandey 2003).

Finally, the third construct, *performance*-*based self*-*esteem*, represents a contingent form of self-esteem, indicating that the individual’s feeling of being a valuable person depends on his/her accomplishments within the work domain (Hallsten et al. 2005). Typically, individuals with high performance-based self-esteem have a strong need to prove their competence in order to feel worthy. As failures and setbacks are particularly detrimental to the self-esteem of these individuals, they put great effort into performing well and strive constantly for success (Hallsten et al. 2005). The constant risk of not succeeding puts these individuals under great stress because they experience a threat to their self-worth as failure would be tantamount to not being good enough. As a consequence, the individual is exposed to a higher risk for negative health outcomes (Blom 2011; Johnson 1997; Johnson and Forsman 1995). Indeed, performance-based self-esteem has been related to cognitive stress symptoms (Albertsen et al. 2010), burnout (Dahlin et al. 2007; Rudman and Gustavsson 2010) as well as sickness presenteeism (Löve et al. 2010).

All three of the described constructs have been associated with tremendous negative individual, work organizational and societal consequences. As far as we know, previous studies have only investigated the relations between the constructs in a pairwise manner and investigations of the relations between all three constructs are lacking so far. In the following section, the hitherto found pairwise relations between the constructs are described in more detail.

Previous research has shown an effect of work–family conflict on emotional exhaustion and burnout (Hall et al. 2010; Karatepe and Tekinkus 2006; Leineweber et al. 2012), but also a relationship in the opposite direction with emotional exhaustion leading to subsequent work–family conflict has been reported (Kelloway et al. 1999; Thompson et al. 2005; Westman et al. 2004). In addition to those two potential causal pathways, there is only a limited number of studies investigating causal and reversed relations simultaneously (e.g. Demerouti et al. 2004; Hall et al. 2010; Steinmetz et al. 2008). One exception is the study reported by Demerouti et al. (2004), which tested the reciprocal relationship of work–family conflict, emotional exhaustion and work pressure. They found exhaustion being a determinant of future work–home interference, but also work–home interference being a causal determinant of subsequent exhaustion. However, most studies in the field are cross sectional (Edwards and Rothbard 2002; Grant-Vallone and Donaldson 2001; Greenhaus et al. 2001; Peeters et al. 2005), and as prospective studies are scarce, the direction of the relationship remains unclear.

Only few studies have linked performance-based self-esteem and work–family conflict (e.g. Innstrand et al. 2010). Individuals with high performance-based self-esteem were found to put personal needs aside in order to meet work requirements. They tended, e.g. to attend work also when sick and reduce their lunches or take work home (Hallsten 2005; Hallsten et al. 2005). Also a reversed causation between work–family conflict and performance-based self-esteem is not to be excluded. For example, Innstrand et al. (2010) found a bidirectional relationship between work–family conflict and performance-based self-esteem. Employees with high performance-based self-esteem were more vulnerable to work–family conflict and those with work–family conflict showed an increase in performance-based self-esteem. Performance-based self-esteem has been shown to play a crucial role in the development of chronic stress by increasing the risk of psychological and physiological exhaustion (Blom 2011; Hallsten et al. 2002, 2005, 2011; Perski 2006). However, while one longitudinal study found that performance-based self-esteem was related to subsequent burnout (Blom 2012), another longitudinal study could not confirm this association (Dahlin and Runeson 2007). To the best of our knowledge, a reversed causation has not been studied yet.

Theoretically, the relationship between experienced imbalance between work and family demands and emotional exhaustion can be explained through the loss spiral assumption that is posed in the conservation of resources (COR) theory (Hobfoll 1989). According to this theory, a vicious circle with regard to the loss of resources is assumed, which is called the spiral loss hypothesis. Employees who may perceive a loss of resources in one domain (e.g. due to high work demands) are more likely to experience other subsequent resource losses in other domains (e.g. family domain, resulting in work–family conflict). Over time less and less resources become available to deal with potential stressors, which can result in emotional exhaustion. This theory is also suitable to explain the relationship between performance-based self-esteem and work–family conflict. In order to maintain self-esteem, maximum effort and resources (i.e. time and energy) are invested in the work domain, which leads to a depletion of resources that otherwise could have been used in the non-work domain. Conflicts between the work and the family role might be especially stressful for individuals that value and need the work role for their feelings of self-worth (Innstrand et al. 2010). It can be speculated that individuals with high performance-based self-esteem have a need to perform well in both the work and the family sphere, which is likely to increase feelings of stress and deficiency. Stress in turn may lead to feelings of conflict or imbalance. Also in the case of a potential relationship between performance-based self-esteem and emotional exhaustion, the COR theory’s spiral loss hypothesis could provide a useful theoretical explanation. The vulnerability through emotional exhaustion could make employees more sensitive to stress and the striving to maintain their self-worth through achievements in the work domain more dominant, which then increases performance-based self-esteem. Moreover, emotional exhaustion, which makes it harder to accomplish work, might be especially stressful for employees basing their self-esteem mainly on their work performance and evolving feelings of insufficiency might increase striving for success even more.

Although in Sweden the labour market participation is more similar for men and women compared with other European countries (Eurostat 2010), there is still an imbalance in the distribution of family-related responsibilities. Usually, women carry a higher overall workload by taking on a larger proportion of the family duties in addition to their full-time employment (Berntsson et al. 2006; Blau et al. 1997). Surprisingly, only a few studies have empirically tested the gender difference in experienced work–family conflict. In fact, there is still no consensus neither with respect to possible gender differences in the amount of experienced work–family conflict nor in regard to whether women are more prone to negative consequences than men (Eby et al. 2005). While some studies comparing men and women working in similar occupations found that women report more conflict between work and home life than men (Lundberg et al. 1994), others showed that men and women report similar levels of conflict (Emslie et al. 2004; Winslow 2005). Regarding performance-based self-esteem and emotional exhaustion research, results are less ambiguous. In general, women report higher performance-based self-esteem than men (Hallsten et al. 2002) and a meta-analysis showed that women experience somewhat higher emotional exhaustion compared with men (Puranova and Muros 2010).

The aim of the present study was to investigate the relations between work–family conflict, emotional exhaustion and performance-based self-esteem over the course of 2 years in a large Swedish national representative sample of working men and women. Gender differences in the investigated relations were studied.

## Methods

### Data collection and participants

The study population consisted of the participants of the SLOSH (Swedish Longitudinal Occupational Survey of Health) study, a longitudinal cohort survey with focus on the association between work organization, work environment and health (Magnusson Hanson et al. 2008). SLOSH comprises all respondents to the Swedish Work Environment Surveys 2003 (*n* = 9,212) and 2005 (*n* = 9,703), building the main representative cohort of 18,915 individuals, which is representative of the Swedish working population in 2003 and 2005. SLOSH started in 2006 with follow-ups conducted every second year. The participants are followed by means of a postal questionnaire in two versions, one for those ‘gainfully employed’, i.e. those in gainful employment for at least 30 % full time or a version for those who are ‘not gainfully employed’, i.e. those working less or who are outside of the labour force. All data collection is carried out by Statistics Sweden. Both SLOSH and the present study have been approved by the Regional Research Ethics Board in Stockholm.

The present study included those individuals who took part in 2006 (overall response rate 65 %) as well as the 2008 follow-up (*n* = 4,690; 78 % of all participants in time 1) and who were gainfully employed at both occasions (*n* = 3,644). After listwise deletion, 3,387 individuals were included in this study, whereof 1,600 were men and 1,787 were women. The study population had an average age of 47.4 ± 9.5 years. About half of the population (51.3 %) had children living at home. Men had on average a higher income, whereas women had a higher education. Moreover, women showed higher levels of performance-based self-esteem, emotional exhaustion and work–family conflict.

### Measures

Information about age and sex was obtained from register data linked to questionnaire responses by means of the unique ten-digit personal identification numbers in Sweden. Information about the participants’ education (university education vs. no university education) and on children living at home (yes vs. no) was derived from survey data. *Work*-*family conflict* was measured with a single item measure (‘Do the demands placed on you at work interfere with your home and family life?’). Response alternatives ranged from 1 (‘very rarely’) to 5 (‘the whole time’). This measure has been used in several other Swedish studies, where it functioned as a predictor for subjective health, sleep quality and repeated sick-leave spells (Alfredsson et al. 2002; Nylen et al. 2007; Voss et al. 2008). *Emotional exhaustion* was measured by a five-item subscale from the Maslach Burnout Inventory–General Survey (MBI-GS; Maslach et al. 1996). Response options ranged from 1 (‘Every day’) to 5 (‘A few times a year or less/Never’) and were reversed so that high scores indicated higher levels in emotional exhaustion (Cronbach’s alpha T1 and T2 (*α* = .87)). *Performance*-*based self*-*esteem* was measured by a four-item scale by Hallsten et al. (2005). A sample item is ‘My self-esteem is far too dependent on my work achievements’. Response options ranged from 1 (‘fully disagree’) to 5 (‘fully agree’). Higher scores indicated higher performance-based self-esteem (Cronbach’s alpha T1 (*α* = .85) and T2 (*α* = .87)).

### Statistical analysis

To study the cross-lagged relationships between the three constructs, structural equation modelling was used by applying robust maximum-likelihood estimation in LISREL 8.7 (Jöreskog and Sörbom 1996). At each time point, work–family conflict was estimated by one item, emotional exhaustion by five items and performance-based self-esteem by four items. To set the scale of the latent variables, one factor loading per latent variable was fixed.

To ensure that our indicators represented the same construct over time, a longitudinal confirmatory factor analysis was run where several models with increased factorial invariance constraints were compared. First a unconstrained model, where all the paths between indicators and latent variables were specified for the two time points with the same pattern and estimated freely, was tested (Brown 2006; Little et al. 2007). Next, weak factorial invariance was tested by setting the loadings invariant, while the last step contained a test of strong factorial invariance, where additionally the intercepts were specified as invariant (Brown 2006). Results of the longitudinal confirmatory factor analysis give indication if differences over time represent true changes that are not caused by changes in the measurement model (Brown 2006). This pretest allows for more valid conclusions regarding the relations of the tested variables. The comparative fit index (CFI) of the different invariance models was compared in order to determine how well the data fitted the constraint conditions; a difference in CFI of .01 or smaller is acceptable indicating invariance (Cheung and Rensvold 2002). In case measurement invariance over time was supported, the weak factorial invariance constraint was kept in the models while analysing the cross-lagged models for more parsimonious testing (Little and Card 2013).

In order to test the relations between the three constructs over time, four different cross-lagged models were analysed. The item-specific measurement errors were allowed to correlate over time to account for the systematic method variance associated with each indicator (Bollen 1989). To take care of contemporary relations, the constructs were allowed to correlate within time points in all models. In all models, we controlled for age, sex, education and children living at home.

1. First, a stability model with only the auto-regressions of work–family conflict, emotional exhaustion and performance-based self-esteem was estimated (Model 1).
2. In a causal model, in addition to the auto-regressions, three paths were added between work–family conflict T1 and emotional exhaustion T2, as well as between performance-based self-esteem T1 and emotional exhaustion T2 and work–family conflict T2 (Model 2).
3. In a reversed causal model, in addition to the auto-regressions, three paths were specified between emotional exhaustion T1 and work–family conflict T2 and performance-based self-esteem T2, and a path between work–family conflict T1 and performance-based self-esteem T2 (Model 3).
4. Finally, a reciprocal model with all paths from the previous models was specified (Model 4).

To investigate whether men and women differed in the pattern and magnitude of the relations between work–family conflict, emotional exhaustion and performance-based self-esteem, a multiple-group comparison between men and women was made for the best fitting model. This procedure was similar to what was done during the longitudinal CFA where different competing models were compared. In the first model, the measurement model was set invariant for men and women but with freely estimated parameters for the structural model. This was compared to a model where even the parameters of the structural model were set invariance between men and women.

To evaluate model fit, the root mean square error of approximation (RMSEA; Steiger 1990), the standardized root mean square residual (SRMR; Bentler 1995), the CFI (Bentler 1990) and the Akaike information criterion (AIC; Akaike 1987) were used in addition to the chi-square fit statistic. For the evaluation of the model fit, the following approximate cut-off criteria were used: for the RMSEA, values lower than .06 (Hu and Bentler 1999), for the SRMR, values smaller than .10 (Hu and Bentler 1995) and for the CFI, values close to or above .97 (Hu and Bentler 1995). A systematic evaluation of the different models was done by comparing the baseline model (Model 1) to more complex models (Models 2, 3, 4) using the chi-square different test. No statistically significant difference in chi-square indicates that the more parsimonious model explains the data equally well compared to the more complex model with additional paths (Kline 1998). Additionally, the other fit indices were used to choose the final best fitting model.

## Results

**Fig. 1:**
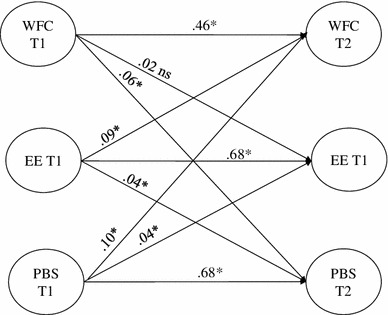
Reciprocal model (Model 4): standardized coefficients. *Notes* *p < .05, dotted line for non-significant path, *WFC* work–family conflict, *EE* emotional exhaustion, *PBS* performance-based self-esteem

In Table 1, descriptive statistics, reliabilities and inter-correlations among all study variables are presented. As can be seen from the table, the reliabilities were acceptable. Overall variables had test–retest reliabilities of at least .46 (see Fig. 1). The highest test–retest reliabilities resulted for emotional exhaustion and performance-based self-esteem. The internal consistencies for all constructs per measurement wave were satisfactory (*α* ≥ .85). In order to provide the basis for testing the relations of emotional exhaustion, work–family conflict and performance-based self-esteem over time, we performed a procedure recommended by Brown (2006) to test for longitudinal invariance. Neither of the steps tested and compared to each other resulted in a CFI difference that exceeded .01. Thus, we can assume that the constructs included in this study are invariant over time (Cheung and Rensvold 2002). In accordance with recommendations from Little and Card (2013), the constraints of weak factorial invariance were maintained for the subsequent testing of our research questions.

**Table 1:** Correlations and descriptive statistics

	M(SD)	1	2	3	4	5	6	7	8	9	10
1. Age	47.40 (10.05)	–									
2. Gender (female)	.53 (–)	.01	–								
3. University education	.37 (–)	−.05*	.13*	–							
4. Having children	.52 (–)	−.30*	−.02	.05*	–						
5. Work–family conflict T1	2.13 (1.04)	−.10*	.05*	.15*	.10*	–					
6. Emotional exhaustion T1	1.63 (1.47)	.00	.12*	.03	−.01	.49*	*.87*				
7. Performance-based self-esteem T1	3.59 (1.44)	−.09*	.05*	.10*	.01	.32*	.32*	*.85*			
8. Work–family conflict T2	2.11 (1.05)	−.13*	.06*	.17*	.12*	.54*	.34*	.27*	–		
9. Emotional exhaustion T2	1.71 (1.46)	−.02	.13*	.04*	−.01	.37*	.67*	.26*	.47*	*.87*	
10. Performance-based self-esteem T2	3.31 (1.40)	−.11*	.06*	.13*	.04*	.30*	.28*	.66*	.31*	.28*	*.87*

Listwise; *n* = 3,387. * *p* < .05; – not applicable. The scales ranged from 1 to 5 except gender (men = 0 and women = 1), age (in years), university education (which was coded 1 = university education, 0 = lower levels of education) and having children living at home (0 = no. 1 = yes). In the diagonal in italic: Cronbach’s alpha

In Table 2, the fit statistics for our four cross-lagged models are shown. All models exhibited significant chi-square values; however, the additional fit indices indicated acceptable fit to the data for all four models.

**Table 2:** Fit statistics for the null and causal model

Models	*df*	*χ* ^2^	RMSEA	SRMR	CFI	AIC	Model comparison	∆*df*	∆*χ* ^2^
Auto-regressions	222	2,294.224*	.0525	.0438	.978	2,450.224			
Causal model	219	2,230.428*	.0521	.0369	.979	2,392.428	1 vs. 2	3	53.80*
Reversed causal model	219	2,231.221*	.0521	.0358	.979	2,393.221	1 vs. 3	3	63.00*
Reciprocal model	216	2,189.406*	.0519	.0334	.979	2,357.406	2 vs. 4	3	51.02*
							3 vs. 4	3	41.82*

* *p* < .05

Comparing the different models (Models 2, 3, 4) to the stability model (Model 1) revealed that all three models show a significant decrease in chi-square, indicating a better fit. Model 4 shows, however, the largest decrease in chi-square (Δ*χ* ^2^ = 104.82, *df* = 6, *p* < .05). In order to test further which of the models is the most parsimonious, these models were compared to each other and Model 4 showed even in comparison with Models 2 (Δ*χ* ^2^ = 51.02, *df* = 3, *p* < .05) and 3 (Δ*χ* ^2^ = 41.82, *df* = 3, *p* < .05), a significant decrease in chi-square. Additionally, this was also confirmed by comparison of the other fit indices (RMSEA, SRMR and AIC; Table 2). Consequently, the reciprocal model (Model 4) was accepted as the best fitting model. Figure 1 shows the reciprocal model and the standardized paths estimates.

In the best fitting model (Model 4), higher levels of work–family conflict at time 1 are associated with performance-based self-esteem (*β* = .06, *p* < .05) at time 2 after control for children, gender, education and age. However, no relationship between work–family conflict at time 1 and emotional exhaustion at time 2 could be established. Emotional exhaustion at time 1 was related to work–family conflict (*β* = .09, *p* < .05) and performance-based self-esteem (*β* = .04, *p* < .05) at time 2. Moreover, performance-based self-esteem at time 1 was related to work–family conflict (*β* = .10, *p* < .05) and emotional exhaustion (*β* = .04, *p* < .05) at time 2.

In addition, some covariates were related to the constructs of interest at time 1, children living at home (*β* = .07, *p* < .05), university education (*β* = .14, *p* < .05) and age (*β* = −.07, *p* < .05) were positively related to work–family conflict; gender (*β* = .05, *p* < .05), university education (*β* = .11, *p* < .05) and age (*β* = −.11, *p* < .05) were related to performance-based self-esteem; and gender was positively related to emotional exhaustion (*β* = .13, *p* < .05).

Further, we tested in the best fitting model whether the structural paths were different for men and women. Multiple-group analysis did not show differences in the relations of the tested constructs over time for men and women (Δ*χ* ^2^ = 87.12, Δ*df* = 21, *p* > .05).

## Discussion

The study had two overall aims; first, we tested the prospective associations between emotional exhaustion, performance-based self-esteem and work–family conflict; secondly, we wanted to investigate possible gender differences in the relations between the three constructs. These relations were tested in a large Swedish national representative sample of working men and women.

Before discussing the main findings of the present study, a few words about the stability of our investigated constructs should be mentioned. All of the three constructs were found to be fairly stable over time. Even though work–family conflict was the least stable of the three constructs, it was found to be rather stable over time with a stability coefficient of .46, which is in line with findings from previous studies such as Rantanen (Rantanen et al. 2012), who found that mean levels of work-to-family conflict were rather stable over such a long time span as 14 years. One explanation could be that contextual factors lead to a perceived imbalance between work and non-work. Those can be difficult to resolve and thus are persistent over time. Even emotional exhaustion which is said to be one of the key aspects of burnout (Maslach et al. 1996) had a high stability over time. An individual who experiences stress over a prolonged period of time gets drained of energy, which eventually results in emotional exhaustion, i.e. feelings of being overextended and depleted of one’s emotional and physical resources. The experience of emotional exhaustion has been associated with a slow recovery even after the energy draining stress source has disappeared. Moreover, individuals might not recognize their need to resolve the stressful situation at once, which eventually leads to even more stress and loss of energy. These facts could explain the stability of this construct in the present study. Performance-based self-esteem and emotional exhaustion were most stable, where about half of the variance of time 2 was predicted by the level at time 1. This is in line with the conceptualization of performance-based self-esteem according to Hallsten et al. (2005), who predicted it to be a habitual pattern that influences behaviour, thoughts and emotions. Still, research has shown that for instance, self-esteem can be affected (Blom 2012; Hallsten et al. 2012; Innstrand et al. 2010).

To proceed with the discussion of the time-lagged relationships, our best fitting model revealed some interesting findings. In contrast to what have been reported from earlier studies (Hall et al. 2010; Karatepe and Tekinkus 2006; Leineweber et al. 2012), we could not establish a relationship between work–family conflict time 1 and emotional exhaustion at time 2. Contrary, we did find that a reversed causal path fitted the data best, where emotional exhaustion preceded work–family conflict. Thus, our results were partly in line with results reported by Leiter and Durup (1996) and Demerouti et al. (2004), who report reciprocal relationships between work–family conflict and emotional exhaustion. Demerouti et al. (2004) conclude that neither work–family conflict nor exhaustion can only be considered cause or effect. One explanation for the missing, but expected, effect of work–family conflict on emotional exhaustion could be that a 2-year time lag might be too long in order to detect a prospective relationship between the constructs. Indeed, the main influence is probably on a daily bases; hence, high values of work–family conflict may lead to contemporary feelings of emotional exhaustion. By allowing constructs to correlate within time, we took care of those contemporary relations. However, our best fitting model showed a statistically significant time-lagged effect from work–family conflict time 1 to performance-based self-esteem time 2. One possible explanation could be that experiencing imbalance between work–family with feelings of conflict and insufficiency in the family under a longer time period implies decreases in self-esteem, for which the individual tries to compensate through maximum effort and performance strivings at work with higher subsequent levels of performance-based self-esteem. The relationship from performance-based self-esteem to work–family conflict is little investigated. To the best of our knowledge, this is one of the first studies investigating the temporal relationship between performance-based self-esteem and work–family conflict. A few studies have investigated the relationship between general self-esteem and work–family conflict, but there are indications that persons with higher self-esteem report lower levels of work–family conflict (Nikandrou et al. 2008). Contrary to performance-based self-esteem, self-esteem can be considered as a resource that helps people to cope with stress. Unfortunately, in the present study, we have no measure of global self-esteem. Therefore, only speculations about this explanation are permitted and future research should investigate this topic further. In line with our findings, one longitudinal study on performance-based self-esteem and work–family conflict found a positive association over time (Innstrand et al. 2012). One potential explanation for this relationship could be that individuals who base their self-worth on work performance tend to put personal needs aside in order to meet their requirements at work. This might interfere negatively with their non-work role as they may prioritize and distribute more time to work issues.

Additionally, we found that emotional exhaustion T1 and performance-based self-esteem T2 were related over time, as were performance-based self-esteem T1 and emotional exhaustion T2. Whereas the relationship from emotional exhaustion to performance-based self-esteem is less established, the relationship between performance-based self-esteem and emotional exhaustion has been found in several other studies (Blom 2011; Hallsten et al. 2002, 2005, 2011; Perski 2006). Indeed, individuals with initial high performance-based self-esteem are said to be more concerned about both their work performance and their accomplishments, which may affect them negatively for instance feeling exhausted. Our finding with relationships in both directions might indicate a vicious cycle with emotional exhaustion increasing performance-based self-esteem and performance-based self-esteem increasing feelings of emotional exhaustion. Consequently, performance-based self-esteem might indeed be not stable but a changeable construct, as previous studies, e.g. Blom (2012) found and we discussed above.

We did not find any differences in gender concerning the relations between the constructs. The national context in which this study was conducted might be one explanatory factor. Compared to other European countries in Sweden, men and women participate approximately to an equal amount in the labour market (women 82 %; men 89 %) and the number of women working full time is increasing (Statistiska Centralbyrån [Statistics Sweden] 2012). Hence, in Sweden, both men and women perceive work–family conflict and are influenced by it to a similar extent, at least in regard to emotional exhaustion. Still, previous reported findings showed a prospective increased risk for emotional exhaustion among both women and men with high work–family conflict, but gender differed in regard to subsequent poor self-rated health and alcohol drinking (Leineweber et al. 2012). Thus, the question whether men’s and women’s health is affected equal or not by work–family conflict concerns further attention.

Our study adds to the existing research by examining different types of plausible causal relationships, thus contributing to a more comprehensive understanding of causality between the three constructs under investigation. Only relatively low regression coefficients were detected. This might, at least partly, be explained by the fact that all constructs showed rather high stability and the auto-regression paths were included in the models. Furthermore, as also constructs were allowed to correlate within time points, a large part of the variability is already explained, and only changes over time are predicted. Still, other unmeasured third variables, such as negative affectivity, social desirability or work load may have affected our results. The solely use of questionnaire data could be seen as a limitation as that might affect our results through common method bias. Also, the conceptualization of work–family conflict is limited in our study; work–family conflict was only assessed by one item. However, the constructs in question in the study are best assessed through using questionnaire data and the measure of work–family conflict is well established (Alfredsson et al. 2002; Nylen et al. 2007; Voss et al. 2008). Future studies should, however, use scales that can capture the different components of work–family conflict (i.e. strain, time and behaviour based) (Greenhaus and Beutell 1985) in order to be able to make more detailed predictions. Even though the time lag of 2 years is a strength, as it allows us to study long-term predictions, it might also be a weakness. Two years might be too long in order to detect effects, especially as work–family conflict might arise on a day-to-day level and therefore also affect performance-based self-esteem and emotional exhaustion on a day-to-day level. Similar problems were detected by Rantanen et al. (2008) when studying work–family conflict and job exhaustion over a 6-year time period. However, to assure that our results are due to actual change over time, we used a parsimonious approach to test longitudinal invariance before testing our models. Furthermore, in contrast to previous studies in the field, which are often conducted within female-dominated work domains, such as professions within the healthcare sector, we used a national representative data set including a wide range of occupations and professions. Hence, gender dominance should be balanced out and results are generalizable to all kinds of occupational groups as well as groups in society.

## Conclusions

Based on our results, we draw the conclusion that the three constructs under investigation were interrelated, which may imply that negative spiral effects can be found between performance-based self-esteem and emotional exhaustion as well as between work–family conflict and performance-based self-esteem. This has an impact on emotional well-being, in such a way that emotional exhaustion might influence health negatively and increase the risk for burnout in the long run. To prevent emotional exhaustion and unhealthy strivings for performance and success in order to increase one’s feelings of self-worth, it seems to be important to reduce the individuals’ perceptions of imbalance between work and non-work.
